# Gutmann’s Donor and Acceptor Numbers for Ionic Liquids and Deep Eutectic Solvents

**DOI:** 10.3389/fchem.2022.861379

**Published:** 2022-03-31

**Authors:** Bruno Sanchez, Paola R. Campodónico, Renato Contreras

**Affiliations:** ^1^ Departamento de Química, Facultad de Ciencias, Universidad de Chile, Santiago, Chile; ^2^ Centro de Química Médica, Instituto de Ciencias e Innovación en Medicina, Facultad de Medicina, Clínica Alemana Universidad Del Desarrollo, Santiago, Chile

**Keywords:** solvent effects, ionic liquids, deep eutectic solvents, anion effect, neoteric solvents, Gutmann numbers

## Abstract

An experimental and computational methodology for the analysis of the Lewis acid/base responses of ionic liquids (ILs) and deep eutectic solvents (DES) is proposed. It is based on the donor and acceptor of the electronic charge ability of Lewis acid and bases concepts (donicity and acceptor numbers, DN and AN, respectively) proposed by Viktor Gutmann. The binding enthalpy between the IL/DES with the probe antimony pentachloride (SbCl_5_) in dichloroethane displays good correlations with experimental data. This approach could serve as a first approximation to predict the responses to H-bonding abilities of new IL or DES. Although useful, the problems encountered to model the electron AN of these solvents limit the usefulness of the approach to completely describe their polarity properties. The experimental data were recorded using UV–Vis spectroscopy for a wide range of ILs and a couple of DES. Two reactions were used as benchmarks to test the reliability of the DN model to discuss the reactivity of real systems in these neoteric solvents.

## 1 Introduction

**FIGURE 1:**
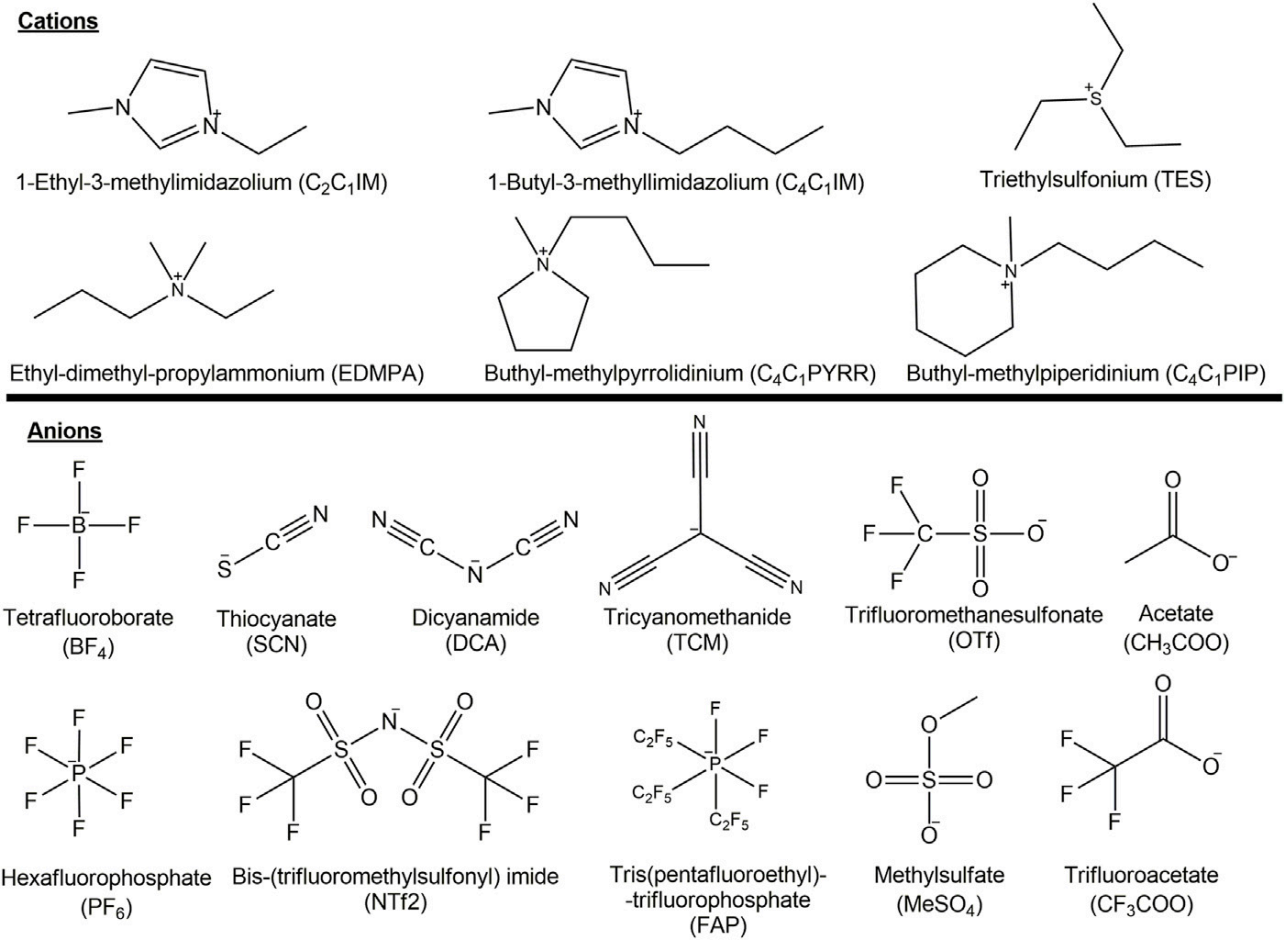
Structures of anions and cations of ionic liquids and the acronyms used in this study.

Ionic liquids (ILs) are defined as organic salts that melt below 100°C (Hallett and Tom, 2011). ILs have been a focus of study in the past decades due to their interesting physical properties such as low vapor pressure (Earle et al., 2006), large electrochemical window (Kazemiabnavi et al., 2016), and catalytic effect over a varied set of chemical reactions (Zhang et al., 2011). However, the principal property that makes them an interesting alternative to conventional solvents is their huge combinatorial flexibility ranging about 10^12^ possible combinations that prompted several authors to propose them as *designer solvents* or *task-specific solvents* (Giernoth, 2010). This flexibility has been applied in diverse areas of research, such as pharmaceutical applications and manufacturing (Zhuang et al., 2021), industrial separation of aromatics (Ayuso et al., 2022), battery electrolyte (Hakim et al., 2021), and cellulose dissolution (Lara–Serrano et al., 2019; Usmani et al., 2020) among many others. Figure 1 shows the acronyms for the different ionic liquids used in this work.

**FIGURE 2:**
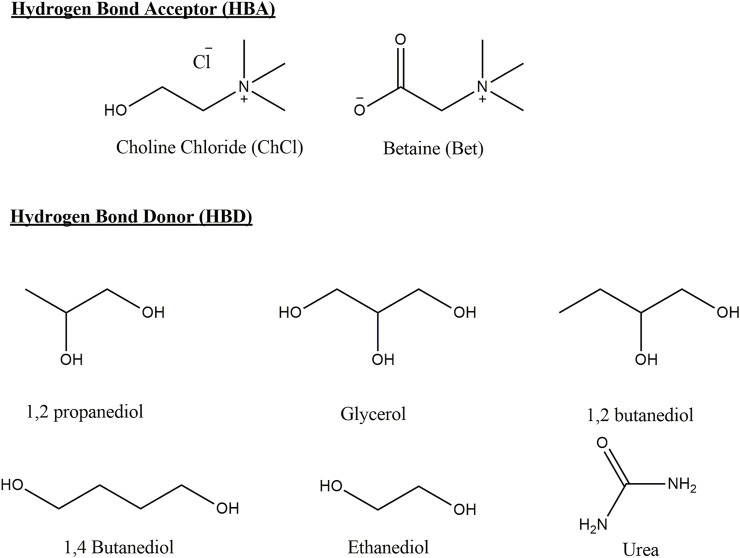
Structures of HBA and HBD of DES and the acronyms used in this study.

On the other hand, deep eutectic solvents (DES) have been perceived as a new class of IL analogs because they share many characteristics and properties with ILs (Zhang et al., 2012). However, it has been recently pointed out that ILs and DESs are two different types of materials. DESs are systems formed from a eutectic mixture of Lewis or Brønsted acids and bases which can contain a variety of anionic and/or cationic species. The classification of DES considers four types of groups based on the nature of the components. The most common DES (type III) studied are formed from the hydrogen bond acceptor (HBA) choline chloride and hydrogen bond donors (HBDs) (Hansen et al., 2021). DES are solvents where the eutectic point temperature is lower to that of an ideal mixture and the mixture remains in the liquid phase at the operating temperatures for a certain composition range (Martins et al., 2019). DES are able to solve the following aspects associated with the first-generation ILs: 1) high cost, 2) presence of impurities, 3) antibacterial activity and toxicity, 4) decomposition towards hydrofluoric or phosphoric acids in water induced by the anions (Płotka-Wasylka et al., 2020), and 5) DES influence on reactions (Körner et al., 2019). DES share some properties with ionic liquids in the sense that they have lower vapor pressures than organic solvents (Dietz et al., 2019) and a high combinatorial flexibility due to the high number of donors and acceptors that allows for the tuning of chemical properties, with the added benefit of being generally cheaper to prepare (using low-cost materials and simpler synthesis and purification methods). These solvents have been proposed for many applications such as in pharmacology, as stabilizers and carriers of active pharmaceutical ingredients (Lu et al., 2016), liquid–liquid extraction and waste disposal (Florindo et al., 2020), and electrolyte for energy storage (Azmi et al., 2022) among others. Figure 2 shows the reagents used to prepare the DES used in this study.

The selection of an appropriate solvent for a given reaction can drastically affect the course of a chemical transformation, so a proper understanding of the solvent properties is needed to predict and rationalize the mechanism involved in the reaction. In this regard, solvent polarity is an important parameter to understand the solute–solvent interactions. However, its loose definition as “*the overall solvation power of a solvent which depends on the sum of all interactions, specific and nonspecific, between the solute and solvent*” (McNaught and Wilkinson 1997) implies that there is not a unique probe or method capable of measuring all aspects of polarity. As a result, several empirical scales of polarity (for both conventional solvents and ionic liquids) have been proposed (Reichardt, 2005; Weingärtner, 2006; Schmeisser et al., 2012), each one with their strengths and drawbacks.

The polarity scale involved in this study is based on the acceptor and donor numbers (AN and DN, respectively), proposed by Gutmann (1978), as the negative of the molar enthalpy for the reaction between the donor SbCl_5_ on a dilute solution of dichloromethane for the DN (Gutmann, 1976) and the chemical ^31^P NMR shift of the triethyl phosphine oxide in the respective pure solvent for the AN (Mayer et al., 1975). An alternative to the calorimetric determination of the DN is the chemical shift of ^23^Na NMR of the NaClO_4_ probe, which gives a good correlation with the thermodynamic measures (Schmeisser et al., 2012) and allows the determination of the DN for a wider range of solvents, including the ILs considered in study. The work of Schmeisser et al. (2012) on the series of [C_2_C_1_im]^+^ ionic liquids it is the most comprehensive source on ILs’ polarity with this technique. However, access to the equipment required to follow this technique is limited in most laboratories. Holzweber et al. (2013) and Lungwitz et al. (2008) proved that the solvatochromic shift of a couple of copper and iron dyes is linearly correlated with the shifts found via RMN and could be used as a way to measure the DN and AN via UV–Vis spectroscopy, which is the methodology followed in this study.

In this context, the evaluation of the Lewis acid/base responses for a series of ILs and DES was carried out in this work to determine the DN and AN descriptors proposed by Gutmann. Two model reactions were used as benchmarks to test the reliability of the DN model to discuss the reactivity of real systems in these neoteric solvents. These reactions have shown a dependence of the reactivity to the donicity of the solvents, so they should be a good indicator to ensure the predictability of the scale in real systems.

## 2 Materials and Methods

### 2.1 Materials

All the reagents used such as choline chloride, betaine, 1,2-propanediol, glycerol, urea, ethylene glycol, 1,2 butanediol, 1,4 butanediol, and ILs were commercially available by Sigma-Aldrich, Merck, and IoLITech. The certificate of analysis guarantees purity ≥99%. DESs were prepared by mixing the corresponding components at the desired mole ratio and heating them for 3 h at 70–80°C until a clear liquid appeared. Previously, the reagents choline chloride, urea, and IL were put under vacuum at 70°C for 3 h to ensure the removal of traces of water before being used. HBD such as 1,2-propanediol, glycerol, and 1,2-butanediol were dried with molecular sieves. After preparation, they were stored in a desiccant prior to being used. Betaine monohydrate was used in the formation of the corresponding DES without further drying.

The probe ferrocyphen was purchased from BOC Science and used without further purifying. The probe Cu(acac)(tmen)^+^ClO_4_⁻ was synthetized using the procedure indicated in the literature (Kuzmina et al., 2017) and purified through hot filtration and recrystallization.

### 2.2 Experimental Measurements

#### 2.2.1 Kinetic Measurements

The studied reactions correspond to 2,4,6-trinitrophenyl phenyl ether (TNPPE) with piperazine and 2-chloro-5-nitro pyrimidine (CNP) and morpholine in ILs and DES as reaction media. These reactions have shown good response of the reactivity to the donicity of the solvent, so they were used to ensure the predictability of the scale in these solvents. The kinetics were carried out spectrophotometrically by means of a diode array spectrophotometer HP 8453 with a recirculating bath, maintaining the temperature at 25 ± 0.1°C (40 ± 0.1°C for some DES). All the reactions were studied under excess amine over the substrate to ensure the pseudo-first-order condition, in which the amine concentrations were at least 10 times greater than the substrate concentration. The reactions were started by injection of a substrate (20 µl) stock solution in acetonitrile (0.0015 M) into the amine solution (1 ml in the spectroscopy cell). The pseudo-first-order constant (*k*_*obs*_) values were spectrophotometrically determined at the wavelengths corresponding to their kinetic products (415 and 380 nm, respectively).

#### 2.2.2 Solvatochromic Shift Measurements

A small amount of the probes (ferrocyphen or Cu(acac)(tmen)^+^ClO_4_⁻) was dissolved in each IL and DES studied. Previously, the probe was homogenized and stabilized for 24 h. The measurements were performed in a diode array spectrophotometer HP 8453 at 25 ± 0.1°C using the spectral software.

#### 2.2.3 Product Analysis

In the studied reactions, the increase of the bands centered in the range of 415–380 nm was observed. It was attributed to the corresponding kinetic reaction products for the studied reactions (Ormazabal–Toledo et al., 2013; Campodónico et al., 2020).

### 2.3 Computational Method

All calculations were performed using the Gaussian 09 package visualized by the Gaussview 5.0 program. Initially, the calculations were made at the M06/6-31+g(d,p) level of theory. Antimony atom was represented by the pseudopotential core LanL2DZ, and all the calculations were performed using the SMD model of implicit solvation to represent the solvent dichloroethane. An optimization of geometries at the length of interaction followed by a frequency analysis was performed to characterize the thermodynamic parameters of the system. These energies were compared to the energy of the probe and anion/IL at a non-interacting distance to get the binding enthalpy. This was carried out in order to account for the error of superposition of bases by using the same Hamiltonian to represent the complete system in both cases. Later, the calculations were redone in different levels of theory, changing the DFT functional (to B3LYP and ωB-97XD) and the basis size (to 6-311+g(2df,2p) for the elements of the first three rows and cc-pVTZ-PP for antimony), to ensure the independence of the results on the basis set and functional used.

## 3 Results and Discussion

### 3.1 Solvatochromic Determination of Gutmann Numbers

Table 1 shows the experimental values of DN and AN determined by the solvatochromic shift of the probes of copper and iron (Cu(acac)(tmen)^+^ClO_4_⁻, ferrocyphen) dissolved in ILs. As expected, most of the ANs fall in a narrow range of variations, or slightly lower when compared to the C_4_C_1_IM cation with the C_2_C_1_IM^+^ analogous. We consider, for instance, the slight reduction when C_4_C_1_IMSCN (AN = 25,6) and C_2_C_1_IMSCN (AN = 27,1) are compared, or the null effect observed after comparing C_4_C_1_IMCF_3_COO (AN = 27,0) with C_2_C_1_IMCF_3_COO (AN = 27,1). Therefore, the increase in the chain length has a little impact on the capacity of the cation to accept electric charge density from a donor. A more significant effect can be observed when the nature of the cation is changed. For example, going from an imidazolium cation such as C_2_C_1_IMDCA (AN = 28,4) to a pyrrolidinium cation C_4_C_1_PYRRDCA (AN = 23,1) with the same counter anion shows a greater decrease in the capacity to accept charge. This fact may be traced to the enhanced ability of imidazolium cation to delocalize electronic charge by the presence of nitrogen atoms and double bonds in comparison to pyrrolidinium cation (see Figure 1).

**TABLE 1:** Solvatochromic shifts of the Fe^+2^ and Cu^+2^ dyes and their respective Gutmann numbers for a series of ionic liquids.

Ionic liquid	λ Fe^+2^ (nm)	AN	λ Cu^+2^ (nm)	DN
C_2_C_1_IM DCA	580,2	28,4	-	-
C_2_C_1_IM SCN	583,5	27,1	764,5	76,2
C_2_C_1_IM NTf_2_	574,8	30,1	-	-
C_2_C_1_IM MeSO_4_	-	-	793,9	71,9
C_2_C_1_IM Ac	591,0	24,2	651,4	45,1
C_2_C_1_IM CF_3_COO	583,5	27,1	685,1	52,5
C_2_C_1_IM TCM	579,8	28,5	617,1	36,8
C_2_C_1_IM MeSO_3_	586,0	26,1	683,0	52,0
C_2_C_1_IM ETSO_4_	587,0	25,7	653,7	45,7
C_2_C_1_IM FAP	575,1	30,4	-	-
C_4_C_1_PYRR DCA	594,0	23,1	646,4	44,0
C_4_C_1_IM BF_4_	584,6	26,7	544,0	15,7
C_4_C_1_IM PF_6_	577,0	29,6	523,8	8,8
C_4_C_1_IM DCA	-	-	646,1	43,9
C_4_C_1_IM SCN	587,4	25,6	730,4	61,3
C_4_C_1_IM OTf	582,4	27,5	594,0	30,7
C_4_C_1_IM NTf_2_	574,3	30,7	550,4	17,8
C_4_C_1_IM MeSO_4_	-	-	798,2	72,5
C_4_C_1_IM Ac	591,4	24,1	678,0	51,0
C_4_C_1_IM CF_3_COO	583,7	27,0	679,0	51,2
C_4_C_1_IM TCM	583,7	27,0	607,8	34,4
C_4_C_1_PYRR NTf_2_	587	25,9	556	19,6
C_4_C_1_PIP NTf_2_	548	25,8	548	17,0
EDMPA NTf_2_	579	28,8	553	18,6
TES NTf_2_	580	28,6	550	17,7

In ILs, the AN is, in general, associated to the cationic component of the IL. To assess the influence of the type of cation on the AN’s responses, the anion NTf_2_⁻ was kept fixed. The cations considered include aromatic heterocyclic ring (imidazolium, AN close to 30, for both chain length); piperidinium and pyrrolidinium cations, both with AN close to 26; quaternary amine (ethyl dimethyl propyl ammonium EDMPA, AN = 28,8); and a sulfonium cation (triethylsulfonium TES, AN = 28,6). The values of AN fall within the range between 25,6 and 30 as shown in Table 1. These values could be either evidence for the low response of the capacity to accept charges in IL or it may be attributable to the probe which could be unsuitable to resolve finer differences of the solvent responses. This fact presents an additional problem when a computational method is used to model the system (IL-probe) because a low variance in the experimental data gives flat curves in the correlation between calculated parameters and experimental data. Then, a careful approach is needed to analyze these data.

On the other hand, the DN is associated with the anion of the ILs. Table 1 shows a significant difference in the values of DN. In this case, the probe chosen has enough sensitivity to give a wide range of values which facilitates the comparison with calculated data. As expected, the presence of an oxygen-containing group or cyanate group increases the value of the DN, while the increase in the side chain length of the imidazolium only has a small decreasing effect on the DN in most of the ILs tested. It is worth noting that several ILs reacted with the probes in such a way that spectrophotometric measures can no longer be used reliably.

### 3.2 Theoretical Gutmann Numbers for ILs

**FIGURE 3:**
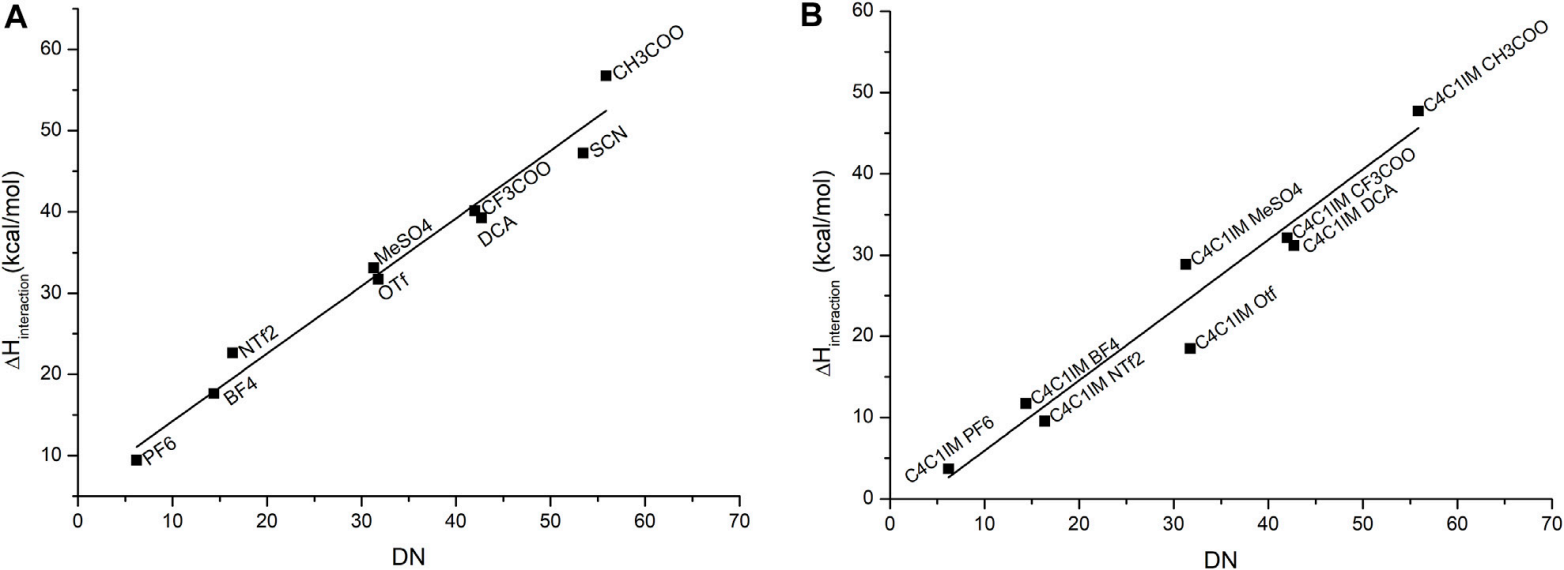
Correlation between the experimental DN values and the calculated binding enthalpies between antimony pentachloride and **(A)** the corresponding anion, where the equation that fits this correlation is y=0,83 DN+5,89 with a correlation coefficient $R^{2}=0,968$ , and **(B)** ionic liquid. The equation that fits this correlation is y=0,86 DN−2,70 with an adjusted R-square value of $R^{2}=0,937$ . The IL C_4_C_1_IMSCN was excluded due to problems of convergence.

To set up a reliable model for the donicity number for ILs, the original definition proposed by Gutmann was slightly adapted to consider the variation in the binding enthalpy of solvent–probe systems, including the presence of the solvent. The calculated results display a qualitative agreement with experimental data. A good linear relationship between them is obtained, as shown in Figure 3.

As it was explained in the methodology section, the geometries of probe–anion and probe–IL pairs were optimized, adding one molecule of dichloroethane as an explicit solvent in the simulation. The stabilization of charges of the anions gave better results which were closely correlated to the experimental data as compared to the same calculation in the gas phase. The choice of solvent was the same as the original work; the focus was to simulate the original experimental conditions in the calculations. However, the computational model treats the IL as a solute which differs from experimental values because in the UV–Vis experiments, the ILs act as solvents. In this case, bulk properties do not appear to influence the donicity of the system, so we conclude that this is good approximation.

All the calculations made supported the original predictions of the change in geometry and coordination number of the antimony atom, going from a trigonal bipyramidal geometry to a more octahedral one. As noted by Gutmann, stronger donors have a smaller antimony–donor distance and longer Sb–Cl distance, as it is shown in Table 2 for the series of anions: MeSO_4_⁻, CF_3_COO⁻, and CH_3_COO⁻. As noted by Gutmann, there is a bigger influence on the Sb–O distance than the Sb–Cl.

**TABLE 2:** Average distance between antimony and chlorine and the distance of antimony and oxygen for a series of similar anions of ILs for the calculated geometries at the M06/6–31+g(d,p) (LanL2DZ for antimony) level of theory.

Anion	Sb–Cl distance (Å)	Sb–O distance (Å)
CH_3_COO⁻	2,390	2,0276
CF_3_COO⁻	2,377	2,0745
MeSO_4_⁻	2,374	2,0948

Table 2 emphasizes a qualitative agreement with Gutmann’s proposal, in the sense that shorter Sb–O distances are in accordance with a more favorable probe–anion interaction. The results for the calculated DN are contrasted against the experimental values for the cases of single anions of the IL and the cation–anion pair in Figure 3. As expected, the major contribution to the DN is given by the anion which gives good correlation with the experimental values. When the cation is added, less dispersion of the data can be observed, thereby suggesting that even at a very low level of approximation (first-order approximation), the probe–anion pair is qualitatively assessed at lower computational cost. It is worth noting that this methodology treats the ILs as an ion pair which is not necessarily true for this kind of solvents, but we believe that, in this specific case, the addition of extra pairs of IL will not change the results drastically.

An additional problem arises when an anion has multiple possible interaction centers that give different local minima configurations. For example, the anion SCN⁻ could interact with the probe through the sulfur atom or through the nitrogen atom, and both configurations seem to be stable enough to generate a minimum in energy. The configuration that gets closer agreement with the experimental values for the DN and, more importantly, that is consistent with the correlation found for the remaining elements of the series is when the molecule interacts through the nitrogen as it has a bigger binding enthalpy than the other configuration and, thus, gets closer to the trend. The choice of the configuration to be used to model the interaction depends on the nature of molecules that surround the anion, since the different polarizabilities of each interaction site are determinant to decide which side the anion is going to prefer to interact with. In this case, both the antimony probe for the calculation and the experimental probe based on copper seem to prefer to interact with the nitrogen side of the anion, but that is not necessarily the case for all solutes.

**FIGURE 4:**
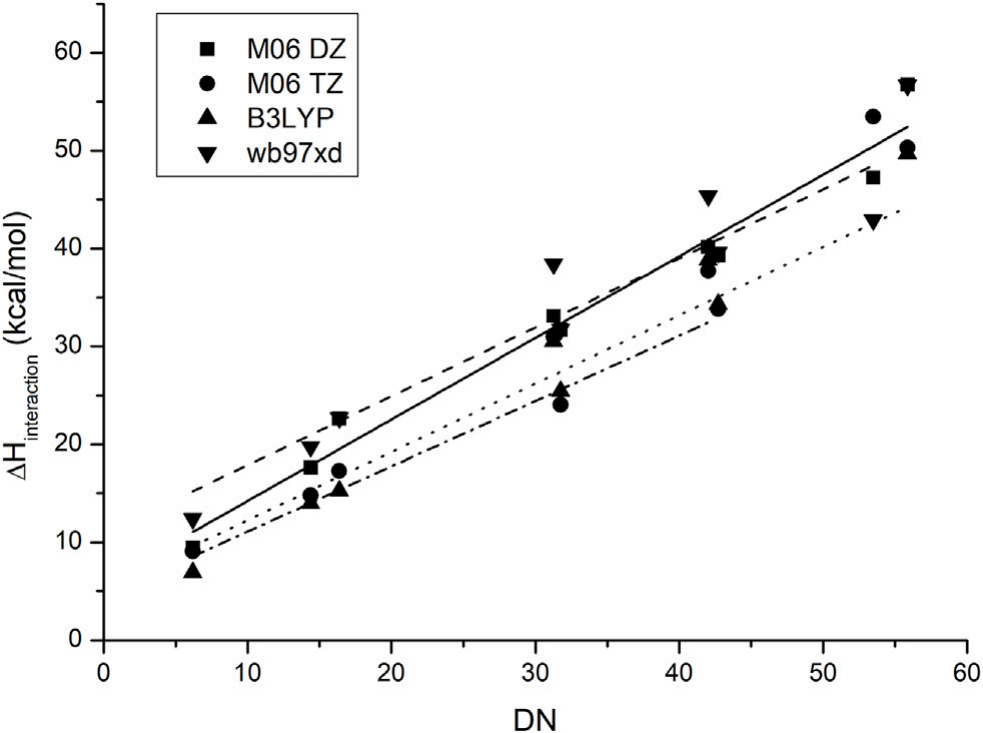
Correlation between the experimental values of the donor numbers and the calculated binding enthalpies between antimony pentachloride and the corresponding ionic liquid when the functional and basis set are changed.

Figure 4 shows the behavior of the anion–probe pair when the functional and the basis set are changed. As observed, although there is a small difference in the absolute values and slopes for each method, the placement of each IL does not change and the same order is predicted for all of them. The increase in base size gives a small improvement in accuracy, thereby lowering the dispersion of the data, yet it is not enough to justify the increase in calculation time that this change originates: a smaller base set with the appropriate functional can give similar results at a lower computational cost.

**FIGURE 5:**
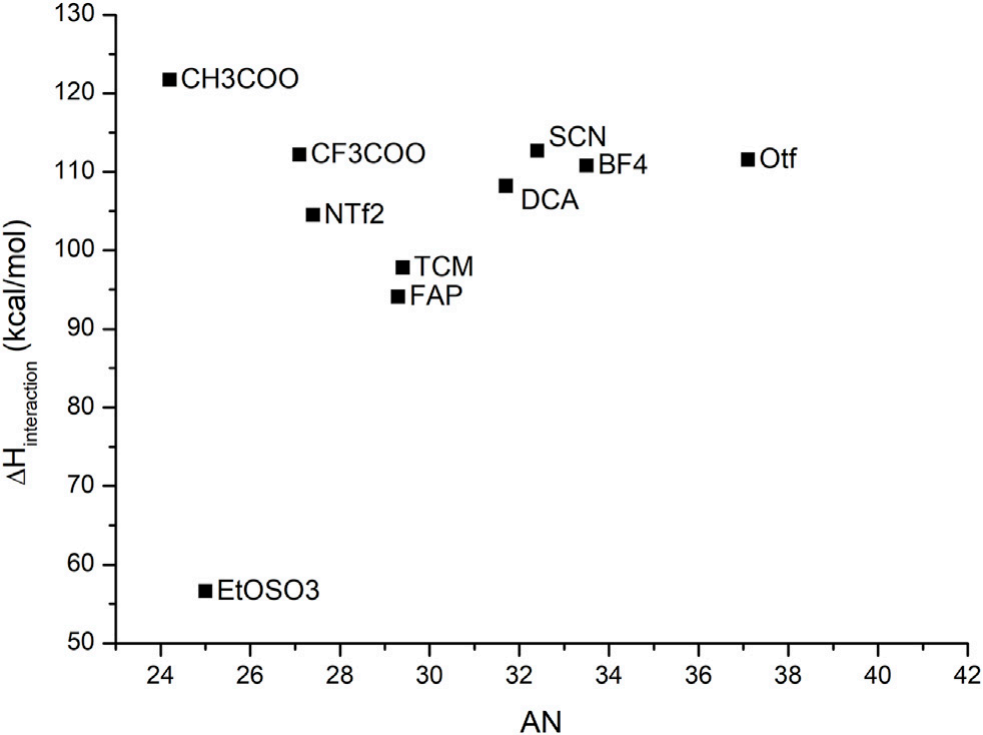
Acceptor number and binding enthalpies between the probe and the IL. The lack of a discernible trend prevents us from considering successful this methodology of calculation.

When a similar approach was attempted to model the AN of ILs, no correlation could be found between the calculated data and the experimental data. Since the acceptor scale proposed by Gutmann was established based on the NMR shift of the phosphorus atom of the probe triethylphosphine oxide, this probe was used in the optimization with each IL. Neither binding enthalpies between the probe and solvent nor RMN shifts of the phosphorous atom displayed good correlations with the experimental data, as shown in Figure 5. This could be traced to the fact that, in contrast to the DN scale, in the AN scale, the IL acts as a solvent in which the probe is dissolved. Since the addition of an implicit solvent in the calculation of the DN parameter improved the correlation with the experimental data, we expected a similar response. This result suggests that the donor and acceptor molecules behave hardly different. We think that a more universal quantum chemical model incorporates second-order effects, including polarizability effects. Work along this line is under development in our group.

### 3.3 Gutmann Numbers for DES

Following a similar approach to determine AN and DN numbers for the ionic liquid, the donicity of DES was determined by UV–Vis spectroscopy. The results are summarized in Table 3. When compared to ILs, DES studied show, in general, higher AN, falling within the range of 30–40, compared to IL series which show variations within the range 20–30. It is also noted that the variability within the series is, in general, low. The DN values for DES also have less variability than the IL examined having a range from 35 to 45, while ILs have a much higher range with values ranging from 8 to 70. This result can be traced to the short series used because it contains only three HBD examined; two of them bearing alcohol groups as the main site of interaction, in contrast to IL where the anion changed within a larger series, varying much more in size and nature of the interacting groups.

**TABLE 3:** Absorption maxima of solvatochromic dyes and the corresponding AN and DN associated with DES at their eutectic ratio at 25°C.

DES	Molar ratio	λ Cu^+**2**^ (nm)	DN	λ Fe^+**2**^ (nm)	AN
ChCl–ethylene glycol	1:2	630	40,1	554	39,1
ChCl–propanediol	1:2	655	45,9	558	37,4
ChCl–glycerol	1:2	615	36,3	546	42,6
ChCl–1,2-butanediol^a^	1:2	646	43,9	563	35,3
ChCl–1,4-butanediol^a^	1:2	-	-	558	37,4
ChCl–urea	1:2	644	43,4	549	41,2
Betaine–ethylene glycol	1:2	618	37,1	558	37,4
Betaine–propanediol	1:2	625	38,8	562	35,7
Betaine–glycerol	1:2	618	37,1	553	39,5
Betaine–1,2-butanediol^a^	1:2	633	40,8	566	34,1
Betaine–urea	1:2	652	45,3	557	37,8

a Measures of the absorption maxima were taken at higher temperatures than 25°C due to the difficulty to maintain the liquid phase stable at lower temperatures.

From Table 3, the following observations are pertinent: the change in HBA from choline chloride to betaine has the effect of lower AN for the betaine-based DES as compared to the corresponding choline-based DES. This result may be traced to the presence of the carboxylate group of betaine because choline has an alcohol group further away in the side chain and a completely detached chlorine ion that can move to accommodate the extra electron density with much more ease than the betaine group can accommodate.

On the other hand, the changes in HBD show that an increase in alcohol groups does not necessarily leads to a higher DN as one could expect, but a decrease as is shown in Table 3 after comparing propanediol and glycerol. On the other hand, the DN remains constant when comparing DES of betaine and ethylene glycol (37,1) and betaine with glycerol (37,1). These inconsistencies could arise from the fact that the probe is measuring the average effect of multiple possible donating centers in the bulk solvent instead of the easily localized negative charges associated with the anion in the case of IL. This effect could put into jeopardy the proposal of this scale as an appropriate measure of the donicity of DES, since little correlation is found when changing the nature of the HBD in a series of similar molecules. A wider exploration of DES with HBD of different types of interacting moieties may show differences of donicity between families of compounds, but at present, the proposed scale clearly offers qualitative and relative criteria but not an absolute and quantitative model for DN index for DES.

For the DES choline chloride–1,2-propanediol, the effect of the proportion between the components and the donicity number of the resulting solvent was also analyzed. The ratio 1:1 could not be measured at room temperature because it took the form of a solution of propylene glycol with undissolved crystals of choline. For the remaining proportions, the results are shown in Table 4. The eutectic ratio 1:2 has higher donicity of the series, and beyond this point, the donicity remains relatively constant, probably due to the saturation of the probe chosen.

**TABLE 4:** Absorption maxima of solvatochromic dyes and the corresponding AN and DN associated for a series of choline chloride–1,2-propanediol at different molar ratios at 25°C.

Molar ratio	λ Cu^+2^ (nm)	DN	λ Fe^+2^ (nm)	AN
1:1	650	44,8	565	34,5
1:2	655	45,9	558	37,4
1:3	633	40,8	554	39,1
1:4	633	40,8	554	39,1
1:5	633	40,8	554	39,1
1,2-Propanediol	582	27,4	550	40,8

**FIGURE 6:**
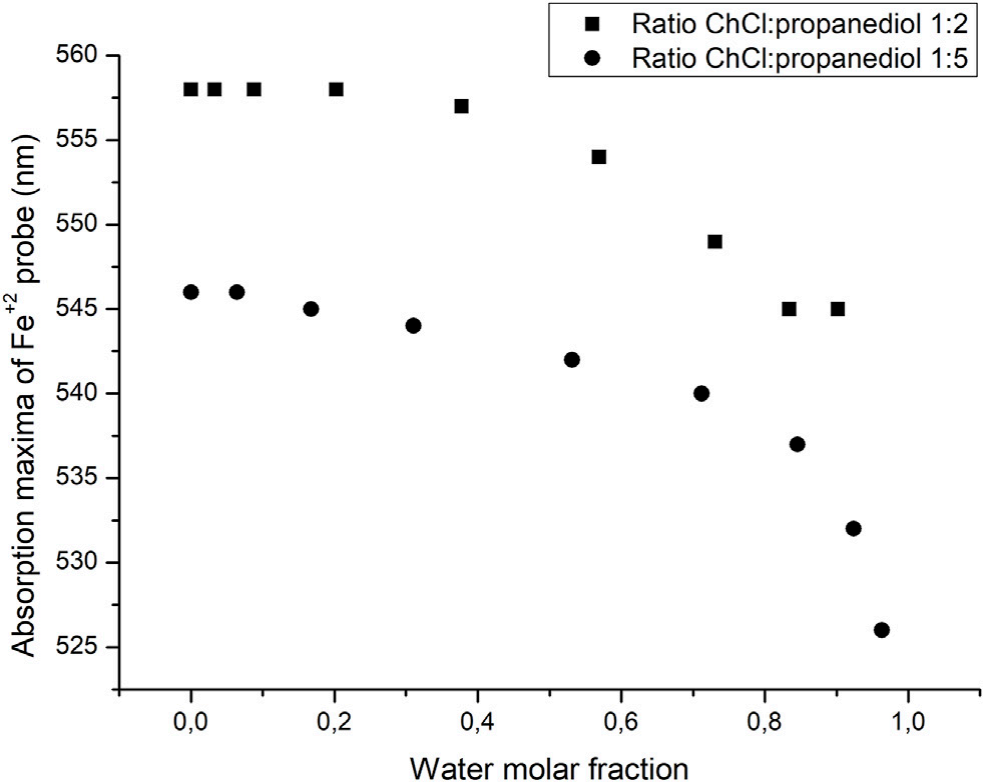
Changes in the absorption maxima of the Fe probe as the molar fraction of water is increased.

A major difference in the approach taken when analyzing the polarity of DES and ILS is the effect that water may have as an impurity in the solvent. Therefore, it is required to evaluate the role that water may have in the polarity of the solvent after increasing water composition. The results are summarized in Figure 6. As it can be seen, when the mole fraction of water is small, the absorption maxima of the probe remains relatively stable being very close to the value of the pure DES, and as the mole fraction of water is increased, both values of the absorption maxima of the probes shifted to lower values, getting away from the normal value of the water-free DES. This result gives a little flexibility in the handling of the DES since even if a little amount of water may be present, the properties of the solvent should not change to a significant extent. Also, since choline is highly hygroscopic, one could reasonably expect to find a small amount of water in this solvent. The variation of light absorption with increasing water content is depicted in Figure 6.

Finally, experimental DN values were compared with theoretical ones, using the binding enthalpy model already applied to IL series. Since DES are usually in a molar ratio of 1:2, this relationship was maintained in the simulation, although a water molecule was not considered in the case of betaine even when the reactive used was a monohydrate. In the IL case, we had a three-component system (cation, anion, and probe) with a clear separation of charges and, therefore, a clear zone of interaction between the probe and the solvent. In the DES case, we have four or five bodies interacting (two HBD molecules, the HBA and the detached chlorine contra anion in the case of choline, and the probe) with the added complexity of a partial charge separation; after all, both choline chloride and betaine have their own electron donor groups that could interact with the probe.

This increase in complexity in the possible configurations that the system could adopt made the previously successful method of calculating binding enthalpies ineffective as it often gave negatives energies and no correlation at all. Since not all possible configurations were explored, it could be the case that a further exploration of the system could show the correlation between the donor number and the binding enthalpy of the system, but it would be more sensible to approach the problem from a different perspective, as is currently being worked by the research team.

### 3.4 Reaction Kinetics

The DN scales obtained for ILs and DES were used to study solvent effects in a model and the reaction between 2,4,6-trinitro phenyl phenyl ether (TNPPE) and a secondary alicyclic amine (piperazine) (Ormazabal–Toledo et al., 2013). S_N_Ar reactions are a good model to analyze solvent effect because these reactions are significantly affected by the reaction media. S_N_Ar involves the stabilization of species associated to the potential energy surface (PES) determining selectivity, reaction rates, and mechanisms (Glossman–Mitnik et al., 2020; Alarcón–Espósito et al., 2016; Newington et al., 2007; D’Anna et al., 2010) Figure 7 shows the accepted mechanism for this S_N_Ar reaction. It occurs in activated aromatic substrates bearing strong electron withdrawing groups (-NO_2_ groups in this case) and a good leaving group (2,4,6-trinitro phenol for this reaction) through an addition–elimination process (Crampton et al., 2004; Terrier 2013). The first step for a stepwise mechanism is the nucleophilic attack to the substrate (*k*_1_ channel in Scheme 3a) leading an anionic σ-adduct named the Meisenheimer complex (MC in Scheme 3a). Then, two processes for its decomposition have been postulated: 1) expulsion of the leaving group (LG) followed a fast proton loss to give the reaction product (*k*_2_ in Scheme 3a) and 2) the base-catalyzed deprotonation of the MC that lost the LG to give the reaction product (*k*_3_ channel in Scheme 3a). The pseudo-first-order rate constant (*k*_obs_) can be expressed as shown in Eq. 1, in which [Nu] represents the concentration of nucleophile.

$$k_{obs}=\frac{k_{2}k_{1}\left[Nu\right]+k_{1}k_{3}{\left[Nu\right]}^{2}}{k_{-1}+k_{2}+k_{3}\left[Nu\right]}$$ (1)

**FIGURE 7:**
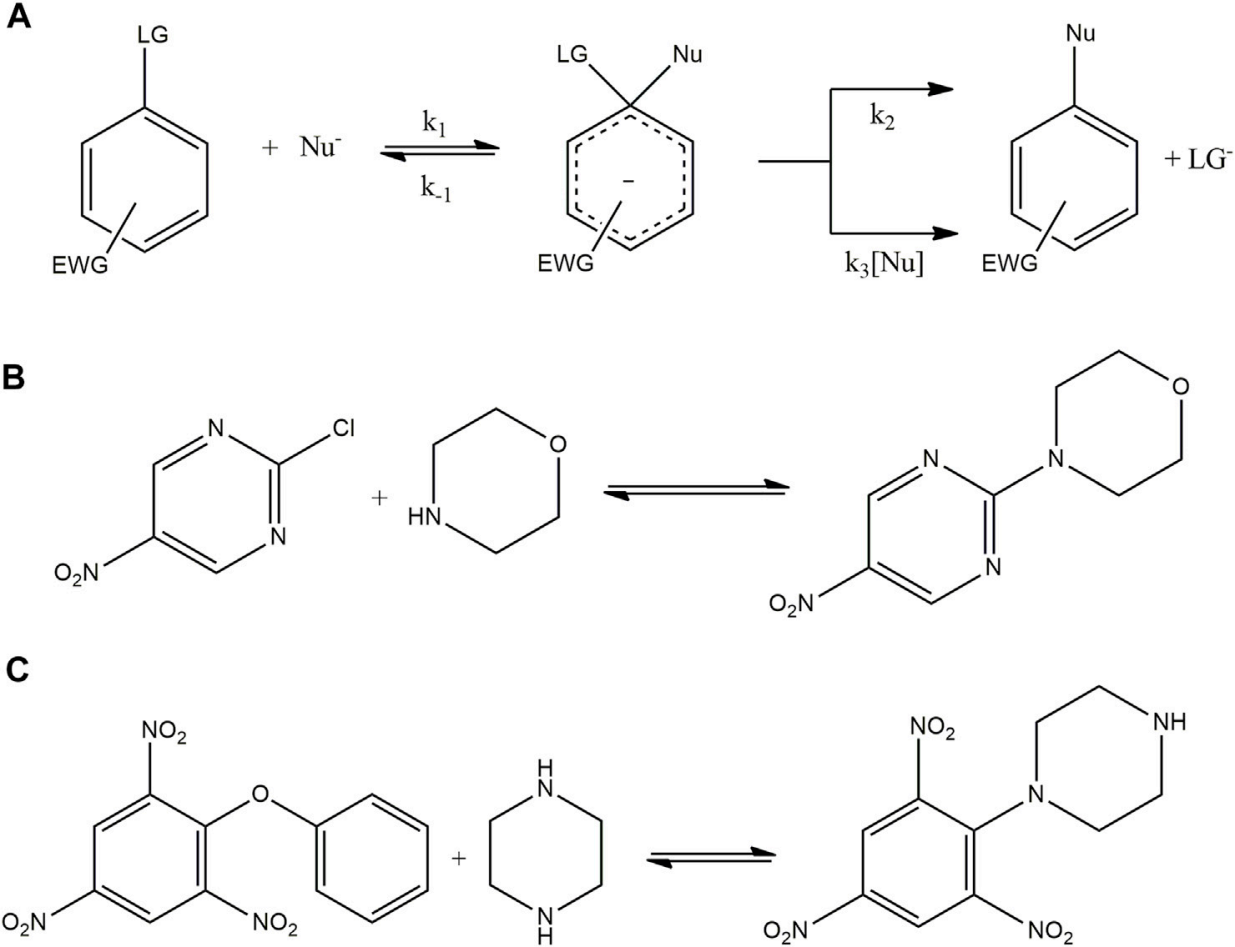
**(A)** General mechanism for a nucleophilic aromatic substitution. The substrate has a leaving group (LG) and some electron withdrawing group (EWG) to favor the substitution. The nucleophile (Nu) attaches to the ring to form the Meisenheimer complex that can generate the desired product through a first-order decomposition (*k*_*2*_) or a second-order decomposition with the help of a second molecule of nucleophile (*k*_*3*_). **(B)** Scheme for the product reaction between CNP and morpholine. **(C)** Scheme for the product reaction between TNPPE and piperazine.

If pathway *k*_*2*_ is faster than *k*_*3*_[Nu], then *k*_*2*_ >> *k*_*3*_[Nu]; therefore, the expression simplifies to

$$k_{obs}=\frac{k_{2}k_{1}\left[Nu\right]}{k_{-1}+k_{2}}$$ (2)

It is noted that the values of *k*_obs_ are in accordance with Eq. 2, and the rate of solvolysis (*k*_0_) and the nucleophilic rate of the reaction (*k*_N_) are obtained as the intercept (*k*_0_) and slope (*k*_N_) of linear plots of the following equation:

$$k_{obs}=k_{N}\left[Nu\right]+k_{0}$$ (3)

The straight lines of these plots in all the solvents tested suggest that the *k*_3_ channel can be discarded as a rate-determining step (RDS) in the reaction mechanism.

The studied reaction in ILs showed a low donicity for C_2_C_1_IM PF_6_ and C_2_C_1_IM BF_4_. In these solvents under the same experimental conditions, no significant amount of product was detected after 3 h. On the other hand, in ILs with high donicity such as C_2_C_1_IM DCA, the solvent established a competitive reaction with the nucleophile for the same reaction site. This fact was evidenced with a change on the reaction product spectra and the kinetic profile, in this case, changing λ_max_ from 416 to 441 nm and changing the color associated to the reaction product. This behavior was not observed in DES in comparison to the ILs studied. In this context, the behavior of the reaction in DES was found to be consistent with the general mechanism for S_N_Ar reaction.

It is noted that this kind of specific interactions between the solvent and reagents cannot be described only by a simple polarity model, so it is important to keep in mind that even when the Gutmann numbers scale can be used to predict reactivity of a system, it only describes one aspect of the interaction between the solvent and solute and no scale and due to the complexity of the possible interactions between the pair, no single scale can describe all the interactions.

Table 5 shows the *k*_N_ values and the molar ratio of the reagents used to prepare the studied DES. It is noted that the donicity trend and the nucleophilic rate coefficients decrease with the increase in the propanediol ratio; however, the constant decrease in the reaction rate even when the DN remained constant, thereby, indicated that the donor capabilities of the solvents are not the only factor that affects the reaction. Since in DES, we have neutral components that have lower nucleophilicity, the problems encountered with IL were not found, yet it is important to indicate that the other DES that were attempted (such choline–urea and choline–glycerol) were not suitable to the kinetic measurements since the high viscosity of the liquid prevented the mixing of the reactants in the cuvette. In the case of choline–glycerol, an increase in temperature decreased enough the viscosity of the solvent to enable the measurement of the reaction rates. However, for choline–urea, even this increase was not enough to overcome the difficulties encountered.

**TABLE 5:** Nucleophilic reaction rates for the reaction between 2,4,5-trinitro phenyl phenyl ether and piperazine in DES.

DES	Molar ratio	Temperature (°C)	k_N_ (M^−1^s^−1^)
ChCl–propanediol	1:2	25	9,42 ± 0,80
ChCl–propanediol	1:3	25	8,14 ± 0,59
ChCl–propanediol	1:4	25	7,31 ± 0,51
ChCl–propanediol	1:5	25	6,48 ± 0,57
ChCl–propanediol	1:2	40	23,24 ± 1,47
ChCl–glycerol	1:2	40	15,21 ± 0,95
ChCl–ethylene glycol	1:2	25	9,70 ± 0,69
Betaine–ethylene glycol	1:2	25	12,28 ± 0,71

Since temperature affects the reaction rate of the system, to compare the rates between propanediol and glycerol, the test with propanediol was repeated at higher temperature in which case we can see that the DES with propanediol has a higher reaction rate than the DES with glycerol. Unfortunately, difficulties in dissolving the solvatochromic dyes in the choline–glycerol mixture prevented us from measuring the Gutmann numbers of this solvent. The viscosity problem in DES is an important issue worth considering. It is noted that the change in the composition of DES (either in the molar ratio of the components or the nature of the HBD and HBA) did have a significant effect on the reactivity derived from the experimental results obtained about the kinetics of these systems. These results cannot be explained by changes in the donicity of the solvent as explained earlier.

**FIGURE 8:**
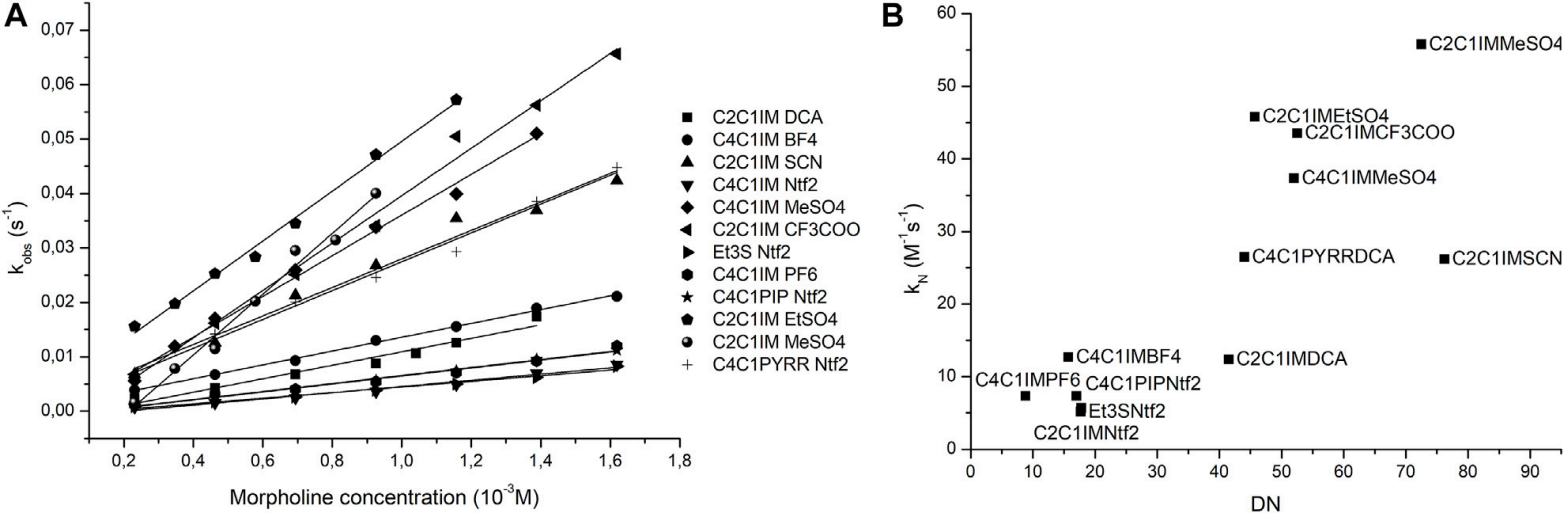
Plots of nucleophile concentration vs. observed pseudo-first-order rate **(A)** and donor number vs. nucleophilic reaction rate **(B)**.

Since the previous reaction was not a suitable model for ionic liquids, another reaction was chosen to prove the prediction capabilities of the donicity model. The reaction between 2-chloro-5-nitro-pyrimidine (CNP) and morpholine was tested in 11 ILs as shown in Table 6. All solvents showed a linear relationship between the nucleophile concentration and the apparent reaction rate that indicates that the mechanism goes through a k_2_ path with no catalysis of a second nucleophile molecule. Since all the solvents had the same reaction mechanism, it is possible to compare them with the donicity of each solvent to see if a trend appears. When plotting the k_N_ of each system against the DN in Figure 8, a general upward trend can be seen where a higher donor number gives a faster reaction rate. The IL C_2_C_1_IM SCN has a reaction rate lower than expected when compared to the remaining elements of the series.

**TABLE 6:** Donor number and nucleophilic rate constant for the reaction between 2-chloro-5-nitropirimidine and morpholine in different ILs.

LI	DN	*k*_*N*_ (M^−1^s^−1^)
C_4_C_1_IM BF_4_	15,7	12,69 ± 0,29
C_4_C_1_IM PF_6_	8,8	7,34 ± 0,50
C_2_C_1_IM DCA	41,5	12,36 ± 1,10
C_2_C_1_IM SCN	76,2	26,19 ± 1,69
C_4_C_1_PYRR DCA	44	26,49 ± 1,10
C_2_C_1_IM Ntf_2_	17,8	5,68 ± 0,41
Et3S Ntf_2_	17,7	5,17 ± 0,33
C_4_C_1_PIP Ntf_2_	17	7,33 ± 0,25
C_4_C_1_IM MeSO_4_	52	37,37 ± 1,36
C_2_C_1_IM MeSO_4_	72,5	55,78 ± 3,01
C_2_C_1_IM EtSO_4_	45,7	45,80 ± 1,53
C_2_C_1_IM CF_3_COO	52,5	43,54 ± 1,80

Note that, in plot 6b), a loose linear relationship could be established, after dropping C_2_C_1_M SCN and C_2_C_1_M DCA points; that coincides with the most polarizable (softer) anions present in the corresponding IL. This result emphasizes the necessity of including second-order effects embodying dipole polarizability contributions within the model.

When the reaction was run in DES (choline chloride with propanediol, glycerol, and urea), the presence of the nucleophile destabilized the solvent, thereby forcing it to precipitate, so no kinetic data could be recorded. This result is a reminder that specific interactions between the solute and solvents are to be incorporated via a super molecule-like approach, and therefore, the implicit third-body effects are important. As a result, a simple model based only on solvent polarity is not suitable enough when dealing with the kinetics of these model reactions.

## 4 Conclusion

An integrated experimental and theoretical study was performed in a series of neoteric solvents, including ionic liquids and deep eutectic solvents. The main message we would send is that a first-order theoretical model, based on the binding enthalpy between probes and solvents, is qualitatively reliable to embody solvent effects within a unified solvation effects model on chemical reactivity of ionic liquids. Second-order effect, incorporating polarizability contributions, should give better responses because of the presence of sizable anions in ILs. This second-order model is, at present, under development in our group. The best predictive capabilities of the donor number scale on a real system using the reaction rates of a S_N_Ar reaction reveals that a high DN solvent leads to higher reaction rates in ILs. It was found that the acceptor and donor capabilities of a DES varies when the molar ratio of the HBD/HBA is modified away from the eutectic ratio but then remains constant for higher ratios. It was also shown that there is a small window of water content in which the polarity properties of the DES remain constant but at higher concentration of water, there is a rapid decrease of the donor/acceptor properties of this new generation of solvents.

## Data Availability

The original contributions presented in the study are included in the article/Supplementary Material, further inquiries can be directed to the corresponding author.
