# Supplementary material for: Gutmann’s Donor and Acceptor Numbers for Ionic Liquids and Deep Eutectic Solvents
Source: Front Chem. 2022 Mar 31;10:861379. doi: 10.3389/fchem.2022.861379 (PMC9008452; doi:10.3389/fchem.2022.861379)
Supplement: Supplementary file 1 [file DataSheet1.pdf]

Supplementary Material

Gutmann´s Donor and Acceptor Numbers for ionic liquids and deep eutectic solvents

Bruno Sanchez[1], Paola R. Campodónico[2*], Renato Contreras[1]

[1]Departamento de Química, Facultad de Ciencias, Universidad de Chile, Casilla 653, Santiago, Chile.

[2]Centro de Química Médica, Instituto de Ciencias e Innovación en Medicina, Facultad de Medicina, Clínica Alemana Universidad del Desarrollo, Chile.

Table 1. Kinetic data for the reaction between TNPPE and piperazine on chloline chloride-1,2propanediol 1:2 at 25°C

[Piperazine] (M)	k_{obs} (s^{-1})
2,70 E-04	7,49 E-03
5,40 E-04	1,08 E-02
1,08 E-03	1,63 E-02
1,35 E-03	2,02 E-02
1,62 E-03	2,38 E-02
2,16 E-03	2,73 E-02
2,70 E-03	2,94 E-02

Table 2. Kinetic data for the reaction between TNPPE and piperazine on chloline chloride-1,2propanediol 1:3 at 25°C

[Piperazine] (M)	k_{obs} (s^{-1})
2,80 E-04	4,05 E-03
5,60 E-04	9,43 E-03
8,40 E-04	1,20 E-02
1,12 E-03	1,34 E-02
1,40 E-03	1,49 E-02
1,68 E-03	1,96 E-02
1,96 E-03	2,23 E-02
2,68 E-03	2,83 E-02
3,35 E-04	9,88 E-03
2,68 E-03	2,90 E-02
2,01 E-03	2,61 E-02
9,72 E-04	1,77 E-02
1,94 E-03	1,99 E-02
3,89 E-03	3,50 E-02
3,24 E-04	8,61 E-03
6,48 E-04	1,29 E-02
1,30 E-03	1,83 E-02
1,62 E-03	2,28 E-02

Table 3. Kinetic data for the reaction between TNPPE and piperazine on chloline chloride-1,2propanediol 1:4 at 25°C

[Piperazine] (M)	k_{obs} (s^{-1})
4,30 E-04	7,16 E-03
8,60 E-04	1,31 E-02
1,29 E-03	1,65 E-02
1,72 E-03	1,87 E-02
2,15 E-03	2,00 E-02
2,58 E-03	2,11 E-02
3,01 E-03	2,57 E-02
2,40 E-04	7,33 E-03
1,37 E-04	1,73 E-03
4,11 E-04	6,21 E-03
8,22 E-04	1,05 E-02
1,23 E-03	1,51 E-02
1,64 E-03	1,68 E-02
2,06 E-03	1,94 E-02
2,47 E-03	2,27 E-02

Table 4. Kinetic data for the reaction between TNPPE and piperazine on chloline chloride-1,2propanediol 1:5 at 25°C

[Piperazine] (M)	k_{obs} (s^{-1})
4,92 E-04	7,43 E-03
9,84 E-04	1,29 E-02
1,48 E-03	1,55 E-02
1,97 E-03	1,90 E-02
2,46 E-03	2,16 E-02
1,70 E-04	2,54 E-03
5,10 E-04	6,98 E-03
1,02 E-03	1,14 E-02
1,53 E-03	1,49 E-02
2,04 E-03	1,74 E-02
2,55 E-03	1,97 E-02
3,06 E-03	2,15 E-02

Table 5. Kinetic data for the reaction between TNPPE and piperazine on chloline chloride-1,2propanediol 1:2 at 40°C

[Piperazine] (M)	k_{obs} (s^{-1})
1,93 E-04	5,66 E-03
3,86 E-04	1,32 E-02
5,79 E-04	1,66 E-02
7,72 E-04	2,07 E-02
9,65 E-04	2,60 E-02
1,16 E-03	2,74 E-02
1,35 E-03	3,49 E-02

Table 6. Kinetic data for the reaction between TNPPE and piperazine on chloline chloride-glycerol 1:2 at 40°C

[Piperazine] (M)	k_{obs} (s^{-1})
6,32 E-04	1,07 E-02
9,48 E-04	1,01 E-02
1,26 E-03	2,21 E-02
1,90 E-03	3,12 E-02
2,53 E-03	3,97 E-02
3,16 E-03	4,44 E-02
4,42 E-03	6,85 E-02

Table 7. Kinetic data for the reaction between TNPPE and piperazine on chloline chloride-ethyleneglycol 1:2 at 25°C

**[Piperazine] (M)**	**k_{obs} (s^{-1})**
8,64 E-04	7,18 E-03
1,30 E-03	1,04 E-02
1,73 E-03	1,51 E-02
2,59 E-03	2,15 E-02
3,02 E-03	2,62 E-02
3,46 E-03	3,42 E-02
1,73 E-03	1,71 E-02
2,16 E-03	1,97 E-02

Table 8. Kinetic data for the reaction between TNPPE and piperazine on betaine-ethyleneglycol 1:2 at 25°C

**[Piperazine] (M)**	**k_{obs} (s^{-1})**
4,69 E-04	8,67 E-04
9,38 E-04	3,57 E-03
1,41 E-03	9,96 E-03
1,88 E-03	1,66 E-02
2,35 E-03	2,33 E-02
2,81 E-03	2,56 E-02
3,28 E-03	3,55 E-02

Table 9. Kinetic data for the reaction between CNP and morpholine on C_2C_1IM DCN at 25°C

**[Morpholine] (M)**	k_{obs} (s^{-1})
2,31 E-04	2,40 E-03
4,63 E-04	4,29 E-03
6,94 E-04	6,75 E-03
9,25 E-04	8,80 E-03
1,04 E-03	1,07 E-02
1,16 E-03	1,26 E-02
1,39 E-03	1,74 E-02

Table 10. Kinetic data for the reaction between CNP and morpholine on C_4C_1IM BF4 at 25°C

[Morpholine] (M)	k_{obs} (s^{-1})
2,31 E-04	3,92 E-03
4,63 E-04	6,73 E-03
6,94 E-04	9,30 E-03
9,25 E-04	1,30 E-02
1,16 E-03	1,55 E-02
1,39 E-03	1,89 E-02
1,62 E-03	2,11 E-02

Table 11. Kinetic data for the reaction between CNP and morpholine on C_2C_1IM SCN at 25°C

[Morpholine] (M)	k_{obs} (s^{-1})
2,31 E-04	6,76 E-03
4,63 E-04	1,26 E-02
6,94 E-04	2,13 E-02
9,25 E-04	2,68 E-02
1,16 E-03	3,54 E-02
1,39 E-03	3,69 E-02
1,62 E-03	4,24 E-02

Table 12. Kinetic data for the reaction between CNP and morpholine on C_2C_1IM Ntf_2 at 25°C

[Morpholine] (M)	k_{obs} (s^{-1})
2,31 E-04	7,55 E-04
4,63 E-04	1,56 E-03
6,94 E-04	2,45 E-03
9,25 E-04	3,64 E-03
1,16 E-03	4,92 E-03
1,39 E-03	6,99 E-03
1,62 E-03	8,57 E-03

Table 13. Kinetic data for the reaction between CNP and morpholine on C_4C_1IM $MeSO_4$ at 25°C

[Morpholine] (M)	k_{obs} (s^{-1})
2,31 E-04	5,60 E-03
3,47 E-04	1,19 E-02
4,63 E-04	1,71 E-02
6,94 E-04	2,60 E-02
9,25 E-04	3,38 E-02
1,16 E-03	3,99 E-02
1,39 E-03	5,10 E-02

Table 14. Kinetic data for the reaction between CNP and morpholine on C_2C_1IM CF_3COO at 25°C

**[Morpholine] (M)**	k_{obs} (s^{-1})
2,31 E-04	6,80 E-03
4,63 E-04	1,62 E-02
6,94 E-04	2,51 E-02
9,25 E-04	3,41 E-02
1,16 E-03	5,05 E-02
1,39 E-03	5,62 E-02
1,62 E-03	6,56 E-02

Table 15. Kinetic data for the reaction between CNP and morpholine on ET_3S Ntf_2 at 25°C

**[Morpholine] (M)**	k_{obs} (s^{-1})
2,31 E-04	8,70 E-04
4,63 E-04	1,75 E-03
6,94 E-04	2,62 E-03
9,25 E-04	3,85 E-03
1,16 E-03	4,97 E-03
1,39 E-03	6,20 E-03
1,62 E-03	8,28 E-03

Table 16. Kinetic data for the reaction between CNP and morpholine on C_4C_1IM PF_6 at 25°C

**[Morpholine] (M)**	k_{obs} (s^{-1})
2,31 E-04	1,22 E-03
4,63 E-04	3,11 E-03
6,94 E-04	4,06 E-03
9,25 E-04	5,43 E-03
1,16 E-03	7,12 E-03
1,39 E-03	9,22 E-03
1,62 E-03	1,20 E-02

Table 17. Kinetic data for the reaction between CNP and morpholine on C_4C_1PIP Ntf_2 at 25°C

**[Morpholine] (M)**	k_{obs} (s^{-1})
2,31 E-04	1,30 E-03
4,63 E-04	2,42 E-03
6,94 E-04	3,99 E-03
9,25 E-04	5,66 E-03
1,16 E-03	7,45 E-03
1,39 E-03	9,57 E-03
1,62 E-03	1,12 E-02

Table 18. Kinetic data for the reaction between CNP and morpholine on C_2C_1IM $EtSO_4$ at 25°C

[Morpholine] (M)	k_{obs} (s^{-1})
2,31 E-04	1,56 E-02
3,47 E-04	1,98 E-02
4,63 E-04	2,53 E-02
5,78 E-04	2,84 E-02
6,94 E-04	3,45 E-02
9,25 E-04	4,71 E-02
1,16 E-03	5,72 E-02

Table 19. Kinetic data for the reaction between CNP and morpholine on C_2C_1IM $MeSO_4$ at 25°C

[Morpholine] (M)	k_{obs} (s^{-1})
2,31 E-04	1,59 E-03
3,47 E-04	7,83 E-03
4,63 E-04	1,15 E-02
5,78 E-04	2,02 E-02
6,94 E-04	2,95 E-02
8,10 E-04	3,15 E-02
9,25 E-04	4,00 E-02

Table 20. Kinetic data for the reaction between CNP and morpholine on C_4C_1PYRR DCN at 25°C

[Morpholine] (M)	k_{obs} (s^{-1})
2,31 E-04	6,91 E-03
4,63 E-04	1,42 E-02
6,94 E-04	2,01 E-02
9,25 E-04	2,46 E-02
1,16 E-03	2,93 E-02
1,39 E-03	3,85 E-02
1,62 E-03	4,48 E-02
